# Defensive medicine practices as a result of malpractice claims and workplace physical violence: a cross-sectional study from Egypt

**DOI:** 10.1038/s41598-023-47720-6

**Published:** 2023-12-16

**Authors:** Ahmed Arafa, Ahmed Negida, Mohamed Elsheikh, Mohamed Emadeldin, Hesham Hegazi, Shaimaa Senosy

**Affiliations:** 1 Department of Public Health and Community Medicine, Faculty of Medicine, Beni-Suef University, Beni-Suef, Egypt; 2 Department of Neurology, Virginia Commonwealth University, Richmond, VA USA; 3 Faculty of Medicine, Zagazig University, Zagazig, Egypt; 4 Department of Health Informatics, Graduate School of Medicine and Public Health, Kyoto University, Kyoto, Japan; 5 Department of Emergency Medicine and Traumatology, Faculty of Medicine, Tanta University, Tanta, Egypt; 6 Department of Urology, Faculty of Medicine, Beni-Suef University, Beni-Suef, Egypt; 7 Wirral University Teaching Hospital NHS Foundation Trust, Wirral, UK

**Keywords:** Health care, Health services, Public health

## Abstract

Defensive medicine refers to practices motivated mainly by legal rather than medical purposes. Increased healthcare costs, overutilization of medical services, and potential harm to patients from unnecessary procedures are among its drawbacks. We performed this study to assess the prevalence of defensive medicine practices in Egypt and their associations with experiencing malpractice claims and workplace physical violence. We investigated 1797 physicians (68.1% male), with an average age of 36.8 ± 9.1 years, practicing in Egypt between January 14th and February 23rd, 2023. SPSS was used for statistical analysis. The majority reported engaging in defensive medicine practices. Specifically, 89.6% acknowledged avoiding high-risk procedures, 87.8% refrained from treating high-risk patients, 86.8% admitted to making unnecessary referrals, 84.9% acknowledged ordering unnecessary tests, 61.4% reported performing unnecessary procedures, and 56.4% disclosed prescribing unnecessary medications. Obstetricians and surgeons exhibited the highest rates of defensive medicine. Using linear regression analysis adjusted for age and sex, malpractice claims and workplace physical violence were associated with defensive medicine score (zero-100): βs (95% CIs) = 5.05 (3.10, 6.99) and 5.60 (3.50, 7.71), respectively, (*p* values < 0.001). In conclusion, defensive medicine is deeply ingrained in the clinical routines of Egyptian physicians. Establishing a comprehensive national medical liability framework is required.

## Introduction

Defensive medicine refers to the practice of ordering procedures, tests, medications, or referrals (positive defensive medicine) or avoiding high-risk patients or procedures (negative defensive medicine) that are motivated primarily by the need to avoid litigation, rather than by the best interests of the patient^1^. Positive defensive medicine can drive up healthcare costs and potentially harm patients by subjecting them to unnecessary tests and procedures^2,3^. Negative defensive medicine, on the other hand, may hinder high-risk patients from receiving appropriate healthcare^2,3^.

Despite its negative consequences, defensive medicine has become integrated into the clinical practice of physicians worldwide^3–32^. The prevalence of defensive medicine has varied widely across studies. In Japan, a survey involving 171 gastroenterologists revealed that 98% of them acknowledged engaging in defensive medical practices^18^. Among 252 breast pathologists in the United States, 88% reported resorting to defensive medicine^32^. A study involving 204 doctors from the United Kingdom found that 78% of them admitted to practicing some form of defensive medicine^4^. In a survey conducted among 1804 physicians who attended the 2017 Congress of Chinese Obstetricians and Gynecologists Association, 62.9% strongly agreed or agreed with the concept of defensive medicine^17^.

The Egyptian healthcare system is characterized by a combination of public and private healthcare providers, offering a range of services from basic primary care to specialized medical treatments. However, little is known about the magnitude of defensive medicine and its associations in Egypt with only one study investigating a few defensive medicine practices among 261 junior physicians from one hospital in Cairo^33^.

Additionally, malpractice claims have been increasing in Egyptian healthcare settings^34^. Egypt’s legal framework may be perceived as favoring patients in medical malpractice cases. Physicians do not usually receive legal assistance from their healthcare institutions or insurance providers; thus, they might experience a sense of exposure to legal actions^34^. This legal environment can lead physicians to practice defensively to avoid potential legal repercussions.

Furthermore, workplace violence is a concerning issue in healthcare systems worldwide, with incidents ranging from verbal abuse to physical attacks on medical professionals. This violence can have detrimental effects on both the physical and psychological well-being of healthcare workers, impeding their ability to provide essential care and jeopardizing the overall quality of healthcare delivery^35^. Egypt has been witnessing a substantial increase in workplace violence targeting healthcare workers^35–37^. It could be speculated that physicians might protect themselves from workplace violence by resorting to defensive medicine.

In this context, we conducted a nationwide cross-sectional study to investigate the prevalence of positive and negative defensive medicine practices and their associations with experiencing malpractice claims and workplace physical violence among physicians in Egypt. Our hypothesis posited that defensive medicine could be prevalent among physicians practicing in Egypt and that malpractice claims and instances of physical violence in the workplace might be linked to heightened defensive medical practices. Investigating these issues may encourage legal reforms aiming at establishing a medical liability framework in the country.

## Methods

### Study design and setting

In this cross-sectional study, we distributed a survey across several online social media platforms hosting physicians in Egypt. The study took place within the period from the 14th of January to the 23rd of February 2023.

We designed a three-section self-administered questionnaire. The first section included an explanation of the study objectives and eligibility criteria. The second section included the demographic and workplace characteristics. The third section evaluated different defensive medicine practices.

We created the survey using Google Forms (Alphaet Inc. Mountain View, CA, USA). To collect responses, the link was distributed to several social network groups hosting Egyptian physicians such as Facebook and WhatsApp. The Egyptian Medical Syndicate, the main governing body that grants medical licenses to physicians in Egypt, has shared the survey within its social network. Other national professional medical associations and societies shared the questionnaire with their members.

### Participants

This study investigated physicians, regardless of their specialty, position, and seniority, who completed their post-graduation clinical training and were working in public or private hospitals or clinics in Egypt for at least one year at the time of conducting the study.

### Variables

The study variables were physicians’ age, sex, years of experience, specialty, malpractice claims, and workplace physical violence. After reviewing previous literature investigating defensive medicine^3–33^, we designated six questions taking into account that they were (1) covering the main positive and negative defensive medicine practices, (2) relevant to healthcare settings in Egypt, and (3) sufficiently general so they could be applied to nearly all medical specialties. The questions were as follows: “*Do you order tests that are probably not clinically indicated to avoid possible legal consequences ?*”, “*Do you carry out interventions or procedures that are probably unnecessary to avoid possible legal consequences ?*”, “*Do you arrange unnecessary referrals to other specialties to avoid possible legal consequences ?*”, “*Do you prescribe unnecessary medications to avoid possible legal consequences ?*”, “*Do you avoid high-risk patients to avoid possible legal consequences in the case of complications ?*”, and “*Do you avoid high-risk procedures to avoid possible legal consequences in the case of complications ?*”. The possible responses were “*Never*”, “*Rarely*”, “*Sometimes*”, and “*Always*”. While the first four questions assessed the main positive defensive medicine practices, the last two questions assessed the main negative defensive medicine practices.

### Sample size

We calculated the sample size using the Epi-Info version 7 StatCalc, which is available from the Centers for Disease Control (CDC) and the World Health Organization (WHO)^38^. We applied the following criteria for sample size calculation: confidence level (95%), a margin of error (5%), and prevalence of defensive medicine practice (50%)^39^. The minimum number of necessary participants to meet the desired statistical constraints was 385. However, we more than quadrupled the least required sample size to allow for assessing different frequencies of defensive medicine practices.

### Statistical analysis

First, we described the personal and occupational characteristics of physicians and their detailed defensive medicine practices: percentages for categorical variables and mean ± standard deviation for numerical variables.

We calculated Cronbach’s Alpha of reliability for the six defensive medicine questions. Kaiser–Meyer–Olkin (KMO) measure of sampling adequacy, average variance extracted (AVE), and composite reliability (CR) of questions assessing positive and negative defensive medicine practices were calculated.

To compare the overall defensive medicine practices across specialties, we created a defensive medicine score by giving zero, one, two, and three points to the responses “*Never*”, “*Rarely*”, “*Sometimes*”, and “*Always*”, respectively, for every question. Therefore, the points achieved from responding to the six defensive medicine questions ranged between zero and 18. Then, we converted these points into a score ranging between zero and 100 and calculated the mean scores per specialty.

Then, we calculated βs and their 95% confidence intervals (CIs) using linear regressions to detect the associations of age (continuous), sex (men and women), malpractice claims (yes or no), and workplace physical violence (yes or no) with the defensive medicine score. We also applied logistic regression analyses to investigate the associations of malpractice claims and workplace physical violence with the frequency of defensive medicine practices.

Since the variable of years of experience was strongly correlated with age (Pearson correlation = 0.966 and variance inflation factor (VIF) for collinearity = 15.1), we did not include it in our regression models to avoid collinearity.

Data were analyzed using the Statistical Package for Social Science (SPSS) released in 2013 (IBM SPSS Statistics for Windows, Version 22.0, IBM Corporation, Armonk, New York).

### Ethics approval and consent to participate

The study protocol was approved by the Ethics Review Committee of the Faculty of Medicine of Beni-Suef University (FMBSUREC/04012023/Senosy). The study was conducted per the Declaration of Helsinki. Informed consent was obtained from all participants.

## Results

### Characteristics of the study population

A total of 1797 physicians, aged 36.8 ± 9.1 years, participated in this study. Of them, 68.1% were men and 44.6% had work experience > 10 years. Malpractice claims and workplace physical violence were reported by 43.5% and 70.1% of physicians, respectively (Table 1).

**Table 1:** Personal and occupational characteristics of physicians (N = 1797) and their association with defensive medicine score.

Characteristics	Value	β (95% CI)	*P* value
Age (years), mean ± SD	36.8 ± 9.1	− 0.42 (− 0.53, − 0.32)	< 0.001
Men, %	68.1	6.57 (4.50, 8.63)	< 0.001
Malpractice claims, %	43.5	5.05 (3.10, 6.99)	< 0.001
Workplace physical violence, %	70.1	5.60 (3.50, 7.71)	< 0.001

All βs (95% CIs) were adjusted for age and sex.

### Reliability and validity of the questionnaire

The Cronbach's Alpha of reliability for the six defensive medicine questions was 0.787 with 95% CIs ranging between 0.772 and 0.802. The corresponding values for positive and negative defensive medicine were 0.755 (0.736, 0.773) and 0.842 (0.827, 0.856), respectively.

The KMO value stood at 0.751 (*p* value < 0.001). The values of AVE and CR were as follows: 0.548 and 0.829 for the questions assessing positive defensive medicine practices versus 0.821 and 0.902 for the questions assessing negative defensive medicine practices.

### Prevalence of defensive medicine practices

**Figure 1:**
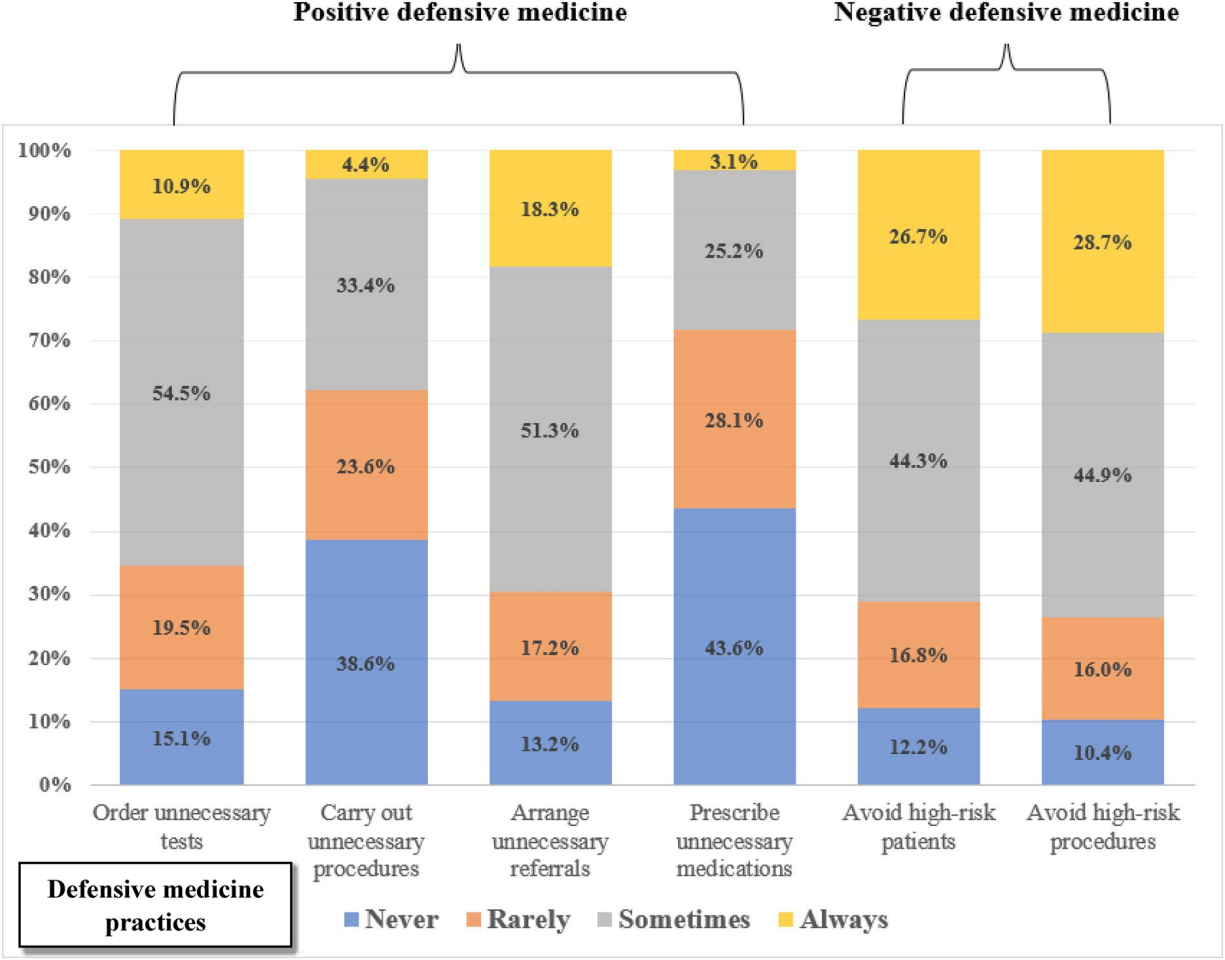
Prevalence of defensive medicine practices among physicians.

Among positive defensive medicine practices, 86.8% of physicians reported arranging unnecessary referrals (17.2% rarely, 51.3% sometimes, and 18.3% always), 84.9% ordering unnecessary tests (19.5% rarely, 54.5% sometimes, and 10.9% always), 61.4% carrying out unnecessary procedures or interventions (23.6% rarely, 33.4% sometimes, and 4.4% always), and 56.4% prescribing unnecessary medications (28.1% rarely, 25.2% sometimes, and 3.1% always). Among negative defensive medicine practices, 89.6% reported avoiding high-risk procedures (16.0% rarely, 44.9% sometimes, and 28.7% always) and 87.8% avoiding high-risk patients (16.8% rarely, 44.3% sometimes, and 26.7% always) (Fig. 1).

**Figure 2:**
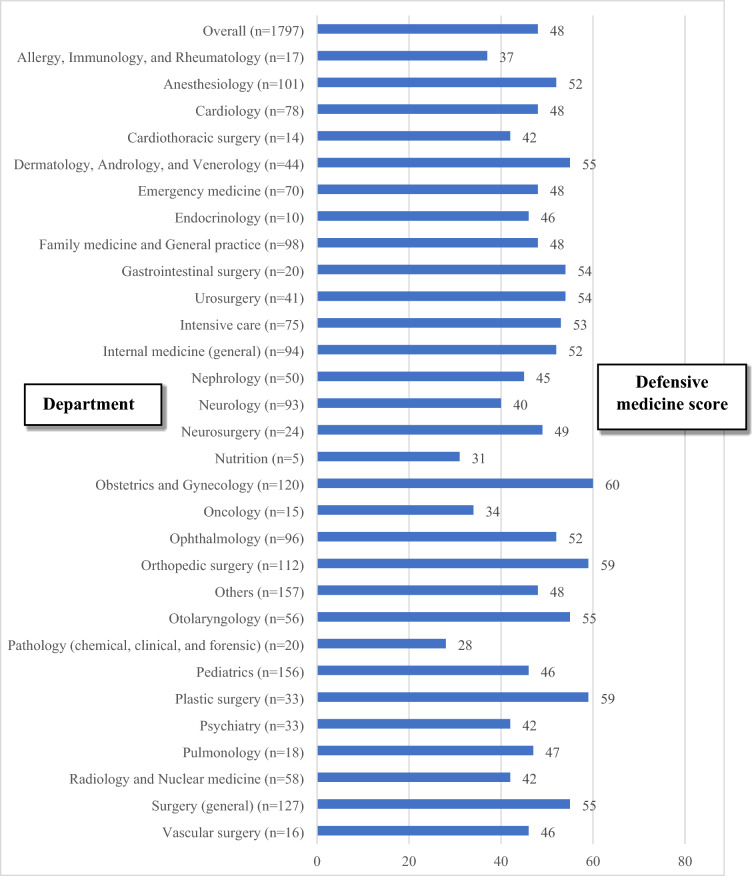
The mean defensive medicine score by specialty on a scale from 0 to 100.

On a scale from zero to 100, the mean defensive medicine score of all physicians was 48. Physicians working in obstetrics, orthopedics, and plastic surgery departments had the highest mean scores (60, 59, and 59, respectively) while those working in pathology and nutrition departments had the lowest mean scores (28 and 31, respectively) (Fig. 2).

### Factors associated with practicing defensive medicine

Age (β = − 0.42, 95% CIs − 0.53, − 0.32), men (β = 0.66, 95% CIs 0.45, 0.86), malpractice claims (β = 5.05, 95% CIs 3.10, 6.99), and workplace physical violence (β = 5.60, 95% CIs 3.50, 7.71) were associated with defensive medicine score (*p* values < 0.001) (Table 1). Additionally, malpractice claims and workplace physical violence were associated with higher frequencies of different defensive medicine practices (Table 2).

**Table 2:** Malpractice claims, workplace physical violence, and the frequency of defensive medicine practices.

Defensive medicine practices	Never	Rarely OR (95% CI)	Sometimes OR (95% CI)	Always OR (95% CI)
Malpractice claims and the frequency of defensive medicine practices				
Order unnecessary tests	1 (Ref)	1.31 (0.93, 1.83)	1.77 (1.32, 2.37)**	2.50 (1.69, 3.70)**
Carry out unnecessary procedures	1 (Ref)	1.42 (1.11, 1.83)**	1.63 (1.30, 2.05)**	2.16 (1.34, 3.47)**
Arrange unnecessary referrals	1 (Ref)	1.09 (0.77, 1.56)	1.29 (0.96, 1.75)	1.90 (1.33, 2.70)**
Prescribe unnecessary medications	1 (Ref)	1.25 (1.00, 1.58)*	1.37 (1.08, 1.74)*	2.06 (1.18, 3.59)**
Avoid high-risk patients	1 (Ref)	0.82 (0.57, 1.17)	1.01 (0.74, 1.37)	1.13 (0.81, 1.57)
Avoid high-risk procedures	1 (Ref)	1.28 (0.87, 1.89)	1.60 (1.14, 2.25)**	1.59 (1.12, 2.27)*
Workplace physical violence and the frequency of defensive medicine practices				
Order unnecessary tests	1 (Ref)	1.19 (0.85, 1.65)	1.75 (1.31, 2.34)**	2.00 (1.31, 3.06)**
Carry out unnecessary procedures	1 (Ref)	1.35 (1.03, 1.76)*	1.64 (1.28, 2.09)**	1.24 (0.74, 2.08)
Arrange unnecessary referrals	1 (Ref)	1.62 (1.13, 2.32)**	1.78 (1.32, 2.40)**	2.33 (1.60, 3.39)**
Prescribe unnecessary medications	1 (Ref)	1.25 (0.98, 1.60)	1.74 (1.33, 2.28)**	1.55 (0.82, 2.96)
Avoid high-risk patients	1 (Ref)	1.67 (1.16, 2.42)**	1.70 (1.24, 2.33)**	1.71 (1.22, 2.41)**
Avoid high-risk procedures	1 (Ref)	1.46 (1.00, 2.16)*	1.63 (1.16, 2.28)**	1.65 (1.16, 2.34)**

All ORs (95% CIs) were adjusted for age and sex.

*Significant at 5%

** Significant at 1%.

## Discussion

Our study indicated a high prevalence of both positive and negative defensive medicine practices among Egyptian physicians. These results underscore the urgency of implementing legal reforms to establish a conducive work environment and a medical liability framework within Egyptian healthcare institutions, aiming to mitigate the practice of defensive medicine.

With several reports from the United States, Canada, Europe, Sudan, China, and Japan, defensive medicine seems to be a pandemic^4–32^. Yet, it is challenging to compare our results with those from previous reports because of the wide variation in defensive medicine definitions, data collection methods, and physicians’ specialties across studies.

We noticed a higher prevalence of negative defensive medicine practices as opposed to positive practices. This trend could potentially be attributed to the relatively youthful age of the participants, with an average age of 36.8 ± 9.1 years. Younger physicians might harbor concerns about their level of experience when it comes to managing high-risk patients or performing procedures with inherent risks, leading them to opt for avoidance as a strategy. This finding could also reflect the idea that, in some cases, inaction might be perceived as a less troublesome course of action than engaging in risky procedures.

Regarding positive defensive medicine practices, the prevalence of carrying out unnecessary procedures or interventions and prescribing unnecessary medications was lower compared to the frequency of arranging unnecessary referrals or ordering unnecessary tests. It is important to note that, unlike referrals and tests, interventions and medications carry a more direct potential for harm to patients, which could expose physicians to malpractice claims.

Among investigated specialties, obstetricians reported the highest frequency of defensive medicine practices. Previous studies came in line and showed increased defensive medicine among obstetricians from Brazil, the United Kingdom, Sudan, Turkey, Israel, and China^12–17^. Interestingly, it is thought that the rising rates of cesarean sections could be attributed to defensive medicine. A study of 403 obstetricians from Brazil showed that obstetricians who had been sued for malpractice performed more defensive cesarean sections than their counterparts who had not been sued^12^. Egypt has one of the highest cesarean section rates in the world. A recent study put the estimate of the cesarean section rate during the period between 2018 to 2020 at 55.1%^40^. Claims of malpractice related to obstetric outcomes, according to the Committee of Medical Ethics of the Egyptian Medical Syndicate, were the most frequently investigated claims^41^. A recent study showed that 28% of 150 examined obstetricians working in Egyptian hospitals were accused of malpractice^34^. Hence, it is probable that the high cesarean section rate in Egypt could be partly explained by defensive medicine. Still, further research is needed to describe the prevalence and determinants of defensive cesarean section in the country.

We noticed that the age of physicians was negatively associated with their defensive medicine practices which agreed with previous findings from the United Kingdom, Italy, and Israel^4,10,30^. Younger physicians, who typically have less experience, may order additional tests or procedures to cover all possible bases and avoid risky situations in order not to make mistakes. Besides, younger physicians may feel pressured by their patients or peers to order unnecessary tests or medications either to protect themselves from potential lawsuits or to provide the best possible healthcare.

In addition, men reported more defensive medicine practices than women. This difference could be explained by the sex distribution across specialties. For cultural reasons, women in Egypt may find it difficult to join high-risk specialties that usually involve more defensive medicine. When we compared the defensive medicine scores between men and women within the same specialty, no significant differences were detected. For example, the defensive medicine mean scores of obstetrics, pediatrics, and family medicine were 60, 47, and 48 in men versus 59, 46, and 48 in women, out of 100, respectively. Besides, malpractice claims and workplace physical violence, which represent risk factors for defensive medicine, were more common among men than women. Therefore, it seems that the decision to practice defensive medicine is not typically gender-specific.

We found that experiencing malpractice claims was positively associated with defensive medicine. Since the fear of litigation is considered the main drive of defensive medicine, this association was quite expected. This association was more robust with positive defensive medicine practices than negative ones. This may reflect a perception among Egyptian physicians that they will not be sued for negative actions.

Our research revealed a positive association between instances of workplace physical violence and the practice of defensive medicine. When physicians face threats of violence or encounter physical harm while delivering care, it can heighten their apprehension and anxiety, leading to an increased tendency to engage in defensive medical practices. Notably, over 70% of the physicians in our study reported experiencing workplace physical violence. While we lack specific information regarding the causes, nature, and perpetrators of these incidents, the elevated rate of violence within the workplace raises questions about the effectiveness of existing laws and protocols governing the physician–patient relationship in Egypt.

Defensive medicine is a complex issue that requires a multifaceted approach to address. In Egypt, three main strategies could be employed to curb defensive medicine practices. First, work overload, inadequate pay, limited equipment, staffing, and medicines, workplace violence, limited access to training and continuing education, and bureaucratic hurdles are issues creating a challenging environment for physicians in Egypt^42^. Under these circumstances, malpractice and adverse events become highly possible^41^ and, as a consequence, defensive medicine becomes heavily practiced. Therefore, improving the work environment in Egyptian healthcare facilities is essential for reducing defensive medicine. Second, Egyptian laws defining medical responsibilities and malpractice are ambiguous with a lack of standardized medicolegal reporting^43^. Typically, physicians in Egypt face lawsuits on a personal basis, with potential consequences including the suspension of their medical license, substantial financial penalties, and the possibility of incarceration. So, legal reforms to address medical responsibility and malpractice are needed to reduce defensive medicine. Healthcare facilities or insurance companies should provide legal consultation for physicians in the case of medical lawsuits^44^. Third, physicians should work on improving communication with patients, educating them about their medical conditions and treatment options, and involving them in their treatment decisions^45^. So, patients can understand the reasoning behind their care and physicians will not be pushed to practice defensive medicine. Unfortunately, it seems that patients in Egypt are not actively integrated into decision-making, and more efforts are needed to address this issue^46^.

Although this study described defensive medicine practices among a large sample of Egyptian physicians representing most specialties, some limitations should be addressed. First, the cross-sectional design restricted our ability to investigate whether defensive medicine practices have been increasing or not; therefore, a longitudinal study is warranted. Second, we relied on an online questionnaire to access physicians. This data collection method could be accompanied by non-response and reporting sorts of bias meaning that the eligible population who were not using social networks frequently were less likely to get the chance to participate in the study, which restricts the study’s generalizability^47^. To minimize this effect, (1) we collected the responses over a period of five weeks, (2) we asked participants to share the survey link privately with their work groups, and (3) we posted the survey on the official Facebook page of the Egyptian Medical Syndicate. Third, since the online questionnaire was shared on different social network sites, we cannot exclude the possibility that some respondents might have filled out the questionnaire more than once. However, none of the responses were duplicated, making this possibility unlikely. Further, we were not able to calculate the response rate for the same reason. Fourth, it could be speculated that physicians who usually practice defensive medicine could have refrained from participation because they were less eager to admit their actions. In contrast, physicians who call for legal reforms might have exaggerated their response to defensive medicine questions. To minimize such forms of bias, we did not ask physicians to unveil their identities or workplaces. Fifth, 68.1% of physicians were men and 77.5% were ≤ 40 years old, suggesting that the prevalence of defensive medicine could be augmented because younger age and male sex were associated with higher odds of defensive medicine practices in this study. Sixth, we did not use a previously validated scale for measuring different defensive medicine practices.

In conclusion, defensive medicine is a prevalent practice within the medical community in Egypt. Our findings suggest that factors such as younger age, past malpractice claims, and incidents of workplace violence are associated with a higher likelihood of engaging in defensive medicine. These results underscore the importance of implementing legal reforms aimed at establishing a comprehensive and transparent medical liability framework. This framework should offer clear definitions of medical responsibilities and malpractice while also ensuring access to legal guidance for physicians. Additionally, it is essential to enact laws that criminalize violence against healthcare professionals.

## Data Availability

Data could be provided by the corresponding author upon a reasonable request.

## References

[1] Tancredi LR, Barondess JA (1978). The problem of defensive medicine. Science.

[2] Vento S, Cainelli F, Vallone A (2018). Defensive medicine: It is time to finally slow down an epidemic. World J. Clin. Cases.

[3] Kakemam E, Arab-Zozani M, Raeissi P, Albelbeisi AH (2022). The occurrence, types, reasons, and mitigation strategies of defensive medicine among physicians: A scoping review. BMC Health Serv. Res..

[4] Ortashi O, Virdee J, Hassan R, Mutrynowski T, Abu-Zidan F (2013). The practice of defensive medicine among hospital doctors in the United Kingdom. BMC Med. Ethics.

[5] Rinaldi C, Leigheb F, Knesse S, Donnarumma C, Kul S, Panella M (2017). Prevalence and costs of defensive medicine: A national survey of Italian physicians. J. Health Serv. Res. Policy.

[6] Ries NM, Jansen J (2021). Physicians' views and experiences of defensive medicine: An international review of empirical research. Health Policy.

[7] Baungaard N, Skovvang PL, Assing Hvidt E, Gerbild H, Kirstine Andersen M, Lykkegaard J (2022). How defensive medicine is defined in European medical literature: a systematic review. BMJ Open.

[8] Vandersteegen T, Marneffe W, Cleemput I, Vandijck D, Vereeck L (2017). The determinants of defensive medicine practices in Belgium. Health Econ. Policy Law.

[9] Tebano G, Dyar OJ, Beovic B, Béraud G, Thilly N, Pulcini C (2018). Defensive medicine among antibiotic stewards: the international ESCMID AntibioLegalMap survey. J. Antimicrob. Chemother..

[10] Asher E, Greenberg-Dotan S, Halevy J, Glick S, Reuveni H (2012). Defensive medicine in Israel: A nationwide survey. PLoS ONE.

[11] Pan M, Rinaldi C, Leigheb F, Donnarumma C, Kul S, Vanhaecht K (2016). The determinants of defensive medicine in Italian hospitals: the impact of being a second victim. Rev. Calid. Asist..

[12] Rudey EL, Leal MDC, Rego G (2021). Defensive medicine and cesarean sections in Brazil. Medicine (Baltimore).

[13] Bourne T, Shah H, Falconieri N, Timmerman D, Lees C, Wright A (2019). Burnout, well-being and defensive medical practice among obstetricians and gynaecologists in the UK: Cross-sectional survey study. BMJ Open.

[14] Küçük M (2018). Defensive medicine among obstetricians and gynaecologists in Turkey. J. Obstet. Gynaecol..

[15] Ali AA, Hummeida ME, Elhassan YA, Nabag WO, Ahmed MA, Adam GK (2016). Concept of defensive medicine and litigation among Sudanese doctors working in obstetrics and gynecology. BMC Med. Ethics.

[16] Asher E, Dvir S, Seidman DS, Greenberg-Dotan S, Kedem A, Sheizaf B (2013). Defensive medicine among obstetricians and gynecologists in tertiary hospitals. PLoS ONE.

[17] Zhu L, Li L, Lang J (2018). The attitudes towards defensive medicine among physicians of obstetrics and gynaecology in China: A questionnaire survey in a national congress. BMJ Open.

[18] Hiyama T, Yoshihara M, Tanaka S, Urabe Y, Ikegami Y, Fukuhara T (2006). Defensive medicine practices among gastroenterologists in Japan. World J. Gastroenterol..

[19] Elli L, Tenca A, Soncini M, Spinzi G, Buscarini E, Conte D (2013). Defensive medicine practices among gastroenterologists in Lombardy: Between lawsuits and the economic crisis. Dig. Liver Dis..

[20] Yan SC, Hulsbergen AFC, Muskens IS, van Dam M, Gormley WB, Broekman MLD (2017). Defensive medicine among neurosurgeons in the Netherlands: A national survey. Acta Neurochir. (Wien).

[21] Yan SC, Hulou MM, Cote DJ, Roytowski D, Rutka JT, Gormley WB (2016). International defensive medicine in neurosurgery: Comparison of Canada, South Africa, and the United States. World Neurosurg..

[22] Cote DJ, Karhade AV, Larsen AM, Castlen JP, Smith TR (2016). Neurosurgical defensive medicine in Texas and Illinois: A tale of 2 states. World Neurosurg..

[23] Solaroglu I, Izci Y, Yeter HG, Metin MM, Keles GE (2014). Health transformation project and defensive medicine practice among neurosurgeons in Turkey. PLoS ONE.

[24] Nahed BV, Babu MA, Smith TR, Heary RF (2012). Malpractice liability and defensive medicine: A national survey of neurosurgeons. PLoS ONE.

[25] Din RS, Yan SC, Cote DJ, Acosta MA, Smith TR (2017). Defensive medicine in U.S. spine neurosurgery. Spine (Phila Pa 1976).

[26] Silberstein E, Shir-Az O, Reuveni H, Krieger Y, Shoham Y, Silberstein T (2016). Defensive medicine among plastic and aesthetic surgeons in Israel. Aesthet. Surg. J..

[27] Sathiyakumar V, Jahangir AA, Mir HR, Obremskey WT, Lee YM, Apfeld JC (2013). The prevalence and costs of defensive medicine among orthopaedic trauma surgeons: A national survey study. J. Orthop. Trauma.

[28] Passmore K, Leung WC (2002). Defensive practice among psychiatrists: A questionnaire survey. Postgrad. Med. J..

[29] Reuveni I, Pelov I, Reuveni H, Bonne O, Canetti L (2017). Cross-sectional survey on defensive practices and defensive behaviours among Israeli psychiatrists. BMJ Open.

[30] Ramella S, Mandoliti G, Trodella L, D'Angelillo RM (2015). The first survey on defensive medicine in radiation oncology. Radiol. Med..

[31] Tanriverdi O, Cay-Senler F, Yavuzsen T, Turhal S, Akman T, Komurcu S (2015). Perspectives and practical applications of medical oncologists on defensive medicine (SYSIPHUS study): A study of the Palliative Care Working Committee of the Turkish Oncology Group (TOG). Med. Oncol..

[32] Reisch LM, Carney PA, Oster NV, Weaver DL, Nelson HD, Frederick PD (2015). Medical malpractice concerns and defensive medicine: A nationwide survey of breast pathologists. Am. J. Clin. Pathol..

[33] Hasan MDA, Shokry DA, Mahmoud RH, Ahmed MM (2021). defensive medicine practice in different specialties among junior physicians in KasrAlAiny Hospitals, Egypt. Indian J. Community Med..

[34] Sobh ZK, Oraby EHA, Abdelaziz SAM (2022). Experience of obstetricians and gynecologists in the management of medicolegal cases in Egypt. BMC Womens Health.

[35] Shukla S (2012). Violence against doctors in Egypt leads to strike action. Lancet.

[36] Arafa A, Shehata A, Youssef M, Senosy S (2022). Violence against healthcare workers during the COVID-19 pandemic: A cross-sectional study from Egypt. Arch. Environ. Occup. Health.

[37] Elsaid NMAB, Ibrahim O, Abdel-Fatah ZF, Hassan HA, Hegazy MH, Anwar MM (2022). Violence against healthcare workers during coronavirus (COVID-19) pandemic in Egypt: A cross-sectional study. Egypt J. Forensic Sci..

[38] StatCalc: Statistical Calculators. Center for Disease Control and Prevention. https://www.cdc.gov/epiinfo/user-guide/statcalc/statcalcintro.html. Accessed 11 Nov 2023.

[39] Khaled Fahim N, Negida A (2018). Sample size calculation guide—part 1: How to calculate the sample size based on the prevalence rate. Adv. J. Emerg. Med..

[40] Wahdan M, Hakim S, El Gaafary M, Sos D, Wassif G, Hussein W (2022). Rising trends in Caesarean section in 6 Egyptian governorates. East Mediterr. Health J..

[41] Azab SMS (2013). Claims of malpractice investigated by the Committee of Medical Ethics, Egyptian Medical Syndicate, Cairo. Egypt J. Forensic Sci..

[42] Kamal Elden NM, Ibrahim Rizk HI, Wahby G (2016). Improving health system in Egypt: Perspectives of physicians. Egypt J. Community Med..

[43] Agarwal R, Gupta A, Gupta S (2019). The impact of tort reform on defensive medicine, quality of care, and physician supply: A systematic review. Health Serv. Res..

[44] Zaki MK, Sobh ZK (2022). Optimum standardization of healthcare medicolegal reports in Egypt: A forensic medicine initiative. Forensic Sci. Int. Rep..

[45] Roodbeen R, Vreke A, Boland G, Rademakers J, van den Muijsenbergh M, Noordman J (2020). Communication and shared decision-making with patients with limited health literacy; helpful strategies, barriers and suggestions for improvement reported by hospital-based palliative care providers. PLoS ONE.

[46] Abdelwahab K, Ibrahim N, Hamdy O, Abdallah A, Zaid A, Shetiwy M (2021). Factors affecting shared decision-making in breast cancer surgeries: Egyptian perspective. Int. J. Cancer Biomed. Res..

[47] Arafa AE, Anzengruber F, Mostafa AM, Navarini AA (2019). Perspectives of online surveys in dermatology. J. Eur. Acad. Dermatol. Venereol..

